# West Nile virus Epidemic in Horses, Tuscany Region, Italy

**DOI:** 10.3201/eid0812.020234

**Published:** 2002-12

**Authors:** Gian Luca Autorino, Antonio Battisti, Vincent Deubel, Giancarlo Ferrari, Riccardo Forletta, Armando Giovannini, Rossella Lelli, Severine Murri, Maria Teresa Scicluna

**Affiliations:** * Istituto Zooprofilattico Sperimentale delle Regioni Lazio e Toscana, Rome, Italy; † Institut Pasteur, Lyon, France; ‡ Istituto Zooprofilattico Sperimentale dell’Abruzzo e del Molise, Teramo, Italy

## Abstract

During the late summer of 1998, veterinary authorities in Tuscany, Italy, received reports of cases of neurologic disease among horses residing in a large wetland area located in the provinces of Florence and Pistoia. West Nile virus was isolated from two of the six horses that died or were euthanized. A retrospective epidemiologic study identified 14 clinical neurologic cases that occurred from August 20 to October 6 (attack rate of 2.8%). A serologic survey conducted over a 700-km2 area in stables with and without apparent clinical cases confirmed a wider spread of the infection, with an overall seroprevalence rate of 38% in the affected area. No significant differences in age-specific prevalence were observed, suggesting that the horses residing in the area had not been exposed previously to West Nile virus and supporting the hypothesis of its introduction in the wetland area during the first half of 1998.

West Nile virus (WNV), named after the district of Uganda where the virus was first isolated in 1937 (1), is a mosquito-borne Flavivirus belonging to the Japanese encephalitis antigenic complex in the family Flaviviridae (2). WNV has been described in Africa, Europe, Middle East, Asia, Oceania (subtype Kunjin), and, more recently, in North America (3).

The ecologic aspects of WNV infection, involving mosquitoes, birds, and humans, were first described in the 1950s in Egypt (4). The agent circulates in nature through continuous enzootic transmission cycles between Culicinae mosquitoes and avian vertebrate hosts and may be introduced into a new territory by migratory birds. Humans and horses are considered incidental hosts. However, an urban cycle with virus amplification by continuous transmission between birds and vectors, and incidentally, humans, has been recently described in Romania and in the United States (5–7).

The infection in humans is usually asymptomatic; however, in 20% of cases, an acute, influenza-like, self-limiting febrile illness may occur, with symptoms of severe encephalitis in <1% of the cases (8). Neurologic disease in both humans and horses was reported for the first time in the late 1950s (9) and 1960s (10,11) respectively, in the Mediterranean area. In the past decade, outbreaks in humans have been reported in Algeria in 1994 (12), in Romania in 1996 and in 1997–1998 (4,13), in the Czech Republic in 1997 (14), in Tunisia in 1997 (H. Triki, pers. comm.), in Russia in 1999 (15), and in birds, humans, and horses in Israel in 2000 (16). In the summer of 1999, the first recorded appearance of WNV in the Western Hemisphere caused fatal disease in humans, horses, and birds in the northeastern United States (5,6,17–19). Outbreaks in horses were described in Morocco from August to mid-October 1996 (20) with 94 cases and 42 deaths, and in France from August to November 2000 (21) with 58 confirmed cases and 20 deaths.

During the late summer in 1998, veterinary practitioners in Italy recorded an increasing number of cases of neurologic disease in horses around an 18,000-km2 wetland area located in the provinces of Florence and Pistoia, known as the Padule del Fucecchio. The area, which covers the central and southern part of the Valdinievole Valley, is home to a number of resident and migratory avian species and is on the route between African wintering areas and European breeding sites of waterfowl, herons, and waders, some of which breed there during the summer.

On the basis of preliminary investigation by public health veterinarians, an outbreak of WNV disease in horses was suspected. On October 19, 1998, the Regional Veterinary Authority banned the movement of Equidae in and out of a 700-km2 area including 20 municipalities. Preliminary epidemiologic and laboratory studies implicated WNV as the etiologic agent, and the ban was lifted 1 month later on November 20, 1998. By this point, the temperature in the area had dropped below levels at which Culicinae mosquito multiplication can occur, and no further cases had been reported since early October. The objectives of our study were to assess whether an epizootic was occurring among horses residing in the area and to perform a cross-sectional serosurvey to gather information about the extension of virus circulation around the wetland area, once the cause of the infection was ascertained.

## Methods

### Retrospective Study

At the end of September 1998, following the initial occurrence of neurologic cases of unknown origin in horses, we initiated a retrospective study to assess possible common exposures. A questionnaire was prepared and distributed to public health veterinarians and veterinary practitioners, who were asked to complete it if they had seen or treated horses during the summer that matched the following case definition: “clinical signs involving the central or peripheral nervous system.” This definition, although broad in scope, was intentionally used to include all possible cases in the course of the investigation. We collected information about location and size of the stable in which the case had occurred; the number, sex, age, and breed of horses present; a checklist of neurologic signs; and the date of onset, duration, and outcome of the clinical course. Additional information requested included whether the horse had received medical treatment within the 15 days preceding the clinical onset and whether any horse had been moved into or out of the stable within 30 days of the date of onset of clinical signs.

During the retrospective study, blood samples were collected in October from 159 horses in the stables where clinical cases had occurred. At an interval of 30 days, a second round of sampling was performed on 124 horses from the same stables. Repeat titers were obtained, from 123 (78%) of the 159 animals bled in October. Further blood samples (n=161) were collected in 16 stables in proximity with those with clinical cases. A confirmed case was defined as a horse with neurologic signs suspect of WNV origin, together with at least one of the following: isolation of WNV, a positive polymerase chain reaction (PCR) assay, and a positive complement fixation (CF) test. A complete necropsy was done on all horses that died during the study, and representative samples of major organs and of the central nervous system were fixed in 10% neutral buffered formalin and processed routinely for histopathology.

### Virology, Pathology, and Serology

Representative portions of the brain and the entire spinal cord were submitted for virologic investigations. Part of the samples were frozen and sent for additional investigations to the Italian National Center for Exotic Diseases (Istituto Zooprofilattico Sperimentale Abruzzo e Molise, Teramo, Italy), the French National Reference Center for Arboviruses and Viral Haemorrhagic Fevers of the Pasteur Institute (Paris, France), and the International Reference Center for Borna Disease (Giessen University, Germany).

Two horses with neurologic signs were euthanized. A sample of cerebrum from one animal (no. 4083V) was analyzed in cell culture and by reverse transcriptase (RT)-PCR and semi-nested PCR. Samples of the cerebellum and the cervical, thorax, lumbar, and sacrum regions of the spinal cord from the second animal (no. 4553V) were also tested. The equivalent of about 0.5 mL of each tissue was mechanically crushed (Minibeadbeater, Biospec Products, Inc., Bartlesville, OK) two times for 30 sec in 2 mL tubes containing 0.5 mL of sterile glass beads and 0.5 mL of Dulbecco’s minimum essential medium (DMEM), supplemented with 10% fetal calf serum (FCS) and antibiotics, and centrifuged at 4,000 rpm for 15 min. The supernatant was diluted 1/10 and 1/100 in the medium and 0.2 mL of the three undiluted and diluted suspensions were incubated 1 hr at 37°C or 28°C in 24-well plates containing Vero E6 cells or Aedes albopictus C6/36 cells. After viral adsorption, cells were washed twice in DMEM and incubated 5 days in DMEM containing 3% FCS. On day 5, cell supernatants were used to infect cell monolayers in 25-cm2 flasks and incubated for 5 additional days. Cells in each well and in the flasks were washed in phosphate-buffered saline (PBS), scraped with a rubber policeman, and deposited on immunofluorescent slides. The cells were fixed by air-drying, the membranes permeabilized in acetone, and dried. The cells were incubated for 30 min at 37°C with an appropriate dilution of hyperimmune mouse ascitic fluids prepared against several arboviruses causing encephalitis in horses. After washing in PBS, the bound antibodies were overlaid with an anti-mouse conjugate labeled with fluorescein. Slides were observed under fluorescent microscope.

RT-PCR and semi-nested PCR were performed on central nervous system samples, using a technique described previously (22). Briefly, total RNA was extracted from a volume of supernatant of ground central nervous system samples. Precipitated RNA was resuspended in diethyl pyrocarbonate–treated distilled water and subjected to RT and PCR, using the oligonucleotide primers (23) WN240 (5´ GAGGTTCTTCAAACTCCAT 3´) and WN312 (5´ GAAAACATCAAGTATGAGG 3´).

One tenth of the incubation mixture was then re-amplified in a semi-nested PCR, using primers WN312 and WNEsn, (5´ CTCCA(T,G)GG(G,C)AGGTT(G,C)AG(G,A)TCCAT 3´). The presence of amplicons of 328 bp and 270 bp, respectively, were examined after 1.5% agarose gel electrophoresis and ethidium bromide staining. The sequences of the amplified products were characterized by sequencing genome position 1402-1656 of the envelope glycoprotein as described (23) by using the alignment algorithm Clustal W.

Serum samples from 12 of the 14 diseased horses were available; the remaining 2 horses had died without having blood samples taken. Additional samples were also available from the same premises where clinical cases had occurred and from nearby stables collected as part of the serologic survey described in the following section. Serum samples were first tested against the major neurotropic viruses infecting horses, including Eastern equine encephalomyelitis, Western equine encephalomyelitis, and Venezuelan equine encephalomyelitis. The presence of antibodies against WNV was evaluated using the CF test and by an immunoglobulin (Ig)G enzyme-linked immunosorbent assay (ELISA) technique.

Reagents for the CF test were supplied by the Onderstepoort Veterinary Institute, South Africa, and the Centers for Disease Control and Prevention, Division of Vector-Borne Infectious Diseases, Fort Collins, Colorado. The CF test assays were performed by the Italian National Center for Exotic Diseases according to the Office International des Epizooties procedures (24). The CF test was considered positive when the sample reacted at a titer of >1:4.

Crude antigens for ELISA were prepared from Vero E6 cells infected with the reference Egyptian Eg101 as described (25). Microtiter plates (Polysorb, Dynatech, Chantilly, VA) were alternately coated with 100 µL of WNV cell lysate antigen and with antigen derived from uninfected Vero cells. Plates were kept overnight at 4°C. Fourfold dilutions of horse sera, starting with 1:100, were placed in the wells. Bound IgG antibody was detected with goat anti-horse IgG conjugated to horseradish peroxidase. H2O2-tetramethylbenzidine was added and the optical density was measured at 420 nm. Serum samples were considered positive for corrected A420 values greater than the corrected mean A420 plus 3 standard deviations of four negative control sera tested at the same dilution.

### Serologic Survey

After the initial notification of neurologic cases in horses, an area consisting of several administrative jurisdictions from which cases had been reported was delineated, and movement of horses in and out of the area was banned. The cause of the illness was not available at that time, and initial clinical and serologic investigations focused on stables located within a 3-km radius of those that had reported clinical cases.

**Figure 1:**
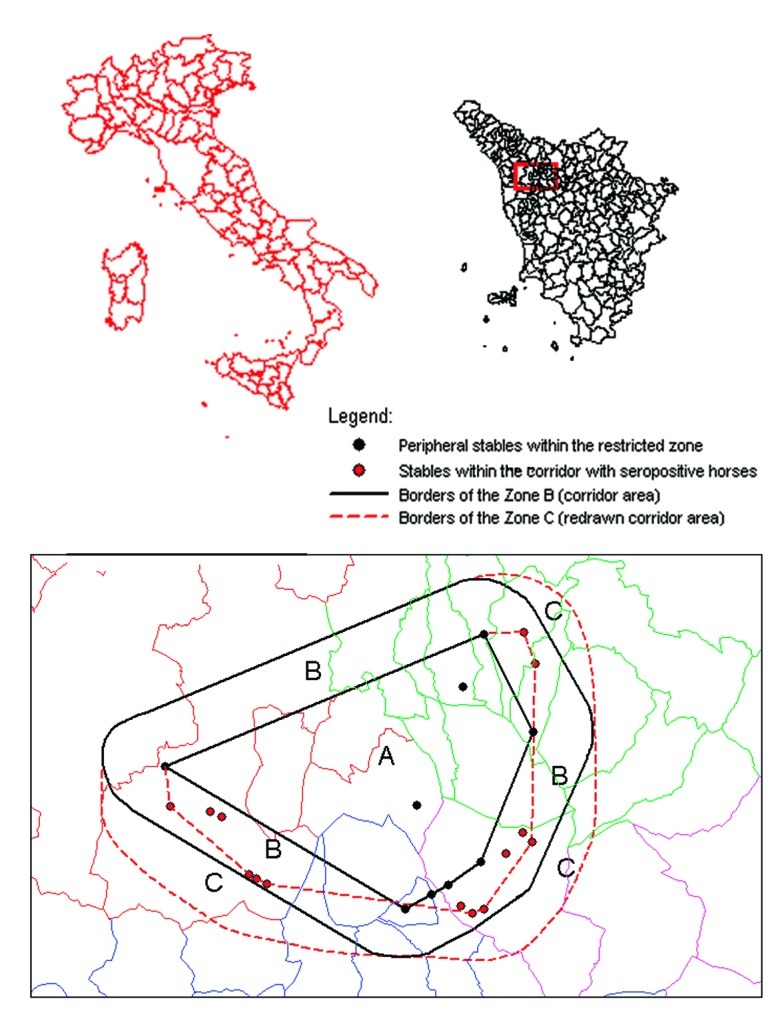
Map of the study area, showing the inner polygon within the restricted zone (zone A), the corridor area (zone B), and the redrawn corridor area (zone C), in the provinces of Firenze, Lucca, and Pistoia, Tuscany Region, Italy.

By November 1998, when WNV was identified as the etiologic agent, we initiated a serosurvey to further define the geographic extent of the infection. The most peripheral stables within the restricted zone were used as reference points to construct a polygonal area whose external angles were all <180° (zone A). As shown in Figure 1, a second polygon was then drawn around the first, with all points 3 km distant from the internal polygon. Thus, a 3-km wide corridor between the first and second polygons was identified (zone B), all stables in this area were investigated, and blood samples from all horses were obtained. After identification of at least one seropositive horse in a stable located within the corridor, the polygon was redrawn with the new positive stable becoming a vertex of a new polygon and the corridor extended an additional 3 km (zone C). A total of 282 horse serologic data (IgG ELISA) were collected. Data were analyzed with the BMDP program version 1.0 (BMDP Statistical Software, Inc., Los Angeles, CA)

## Results

### Pathology

No gross changes were observed in the central nervous system and other organs of the six dead horses. Histologically, a mild-to-moderate nonsuppurative polioencephalomyelitis was present, with consistent involvement of the ventral horns of the thoracic and lumbar spinal cord and of the lower brain stem. Perivascular cuffs of lymphoplasmacytic and histiocytic cells with small and scattered glial nodules and focal gliosis were observed in the gray matter. In some cases, ring and petechial hemorrhages and neuronal degeneration were observed in the lumbar spinal cord (26). These features were highly suggestive of an infection of viral origin.

### Virology

WNV was recovered in the cerebellum (Vero E6 and C6/36) (virus isolate PaAn981) and lumbar part (Vero E6) of the spinal cord of horse no. 4553V. RT-PCR was positive with samples from the cerebellum and the thorax and lumbar regions of the spinal cord. WNV was not isolated from the cerebrum of horse no. 4083V; however, RT-PCR results were positive. Both virus isolation and RT-PCR results were negative from the cerebrum of horse no. 4553V. In all cases, RT-PCR gave a negative result. Laboratory investigations on other viruses affecting horses yielded a negative result.

The sequence of the amplified fragment of 255 bp from the PaAn981 Italian virus RNA (GenBank accession no. AF205883) showed >99% similarity in nucleotide sequence with recent WNV strains responsible for epidemics in Romania in 1996 (GenBank accession no. AF260969), Morocco in 1996 (accession no. AF205884), Kenya in 1998 (accession no. AF146082), Volgograd in 1999 (accession no. AF239988, Israel in 2000 (accession no. AF380669), and France in 2000 (accession no. AF418554) and 98.6% similarity with strains from Israel in 1998 (accession no. AF205882) and New York in 1999 (accession no. AF196835). No change was observed in the amino acid sequence of this short region of the envelope protein.

### Retrospective Study

The questionnaires sent to local veterinarians were returned by the end of October 1998. A total of 14 horses that matched the case definition were identified. They were distributed in nine different stables located in the provinces of Florence, Lucca, Pisa, Pistoia (Figure 1). The first case occurred on August 20 and the last on October 6. Of the 14 horses, 8 recovered, and 2 died,1 and 3 days, respectively, after onset of signs. The remaining four were euthanized because of the severity of the clinical course. The most frequently observed signs were posterior weakness, ataxia, and loss of equilibrium. In the eight animals that recovered, signs persisted for 5–15 days. In three cases, additional clinical signs were observed (mild keratitis, dermal papules, and third eyelid protrusion). Fever was observed in one case, at onset of disease.

Overall, the attack rate in the nine affected stables was 2.8% (14/498, Table 1). The case-fatality rate among horses with neurologic signs was 43% (6/14) including the four horses that were euthanized. No difference in age was detected between fatal cases (horses that died or were euthanized) and those that recovered (Mann-Whitney U Test).

**Table 1:** Complement fixation test findings in stables with clinical cases (zone A)^a^

			Seroprevalence (%)	
Stable	Horses present	Clinical cases	1st sampling^b^	2nd sampling^c^
1	25	2	11/21 (52.4)	not available
2	5	1	3/5 (60)	1/3 (33.3)
3	12	2	6/10 (60)	6/10 (60)
4	49	2	9/13 (69.2)	5/11 (45.4)
5	7	1	2/7 (28.6)	1/5 (20)
6	270	1	5/49 (10.2)	3/41 (7.3)
7	52	1	9/20 (45)	2/20 (10)
8	18	1	6/8 (75)	5/9 (55.5)
9	60	3	12/26 (46.1)	8/25 (32)
Total	498	14	63/159 (39.6)	31/124 (25)

^a^Repeat titers (October–November 1998) were obtained from 123 horses. ^b^Performed October 12–16, 1998. ^c^Performed November 9–13, 1998.

**Figure 2:**
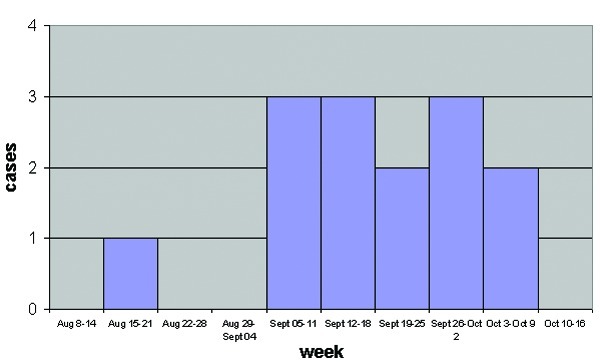
Number of neurologic cases in horses by week of onset, Valdinievole District, Tuscany, Italy, August–October 1998.

The temporal distribution of equine cases by week of illness onset is shown in Figure 2.

On the 12 diseased horses for which blood was available for testing, CF test results were positive for all, with titers ranging from 1:4 to 1:128. In the nine stables from which clinical cases had been notified, 159 of the 498 horses present were bled in October and serologically tested, with 123 of 159 tested a second time 1 month later.

The overall results of the CF test are shown in Table 1. Table 2 shows the results of the first and second round of sampling in the 123 horses that were tested twice. The seroprevalence rate was significantly different between the two samplings (McNemar chi-square test; p<0.01). The 29 horses that were positive at first and second sampling had a geometric mean titer of 1:29 in the first sampling and 1:6 in the second sampling; this difference was statistically significant (Student t test for paired sera on log transformed data, p<0.01). Among the remaining 18 horses that initially tested positive but were negative at second testing, the geometric mean titer of the first sample was 1:15.

**Table 2:** Comparison between first sampling (acute, October 1998) and second sampling (convalescent, November 1998) complement fixation test results in horses from the stables with clinical cases (zone A)

		1st sampling		
		Positive	Negative	Total
2nd sampling	Positive	29	1	30
2nd sampling	Negative	18	75	93
	Total	47	76	123

Serum samples were collected from an additional 161 horses in November 1998 (>3 weeks after the last reported clinical case) from 16 stables located within a 3-km radius from the ones with clinical cases in zone A. Samples were tested first with CF test and later with an IgG ELISA, not available at the beginning of the epidemic. Of the 161 horses, 63/155 (41%) had positive ELISA tests (6 horses were excluded because of inconclusive results) and 30/161 (19%) were CF test–positive with titers ranging from 1:4 to 1:8. The positive horses belonged to nine different premises. The overall seroprevalence rate in these nine stables was 49% (63/129) when the IgG ELISA test was used and 22% (30/134) with the CF test (Table 3).

**Table 3:** Enzyme-linked immunosorbent assay (ELISA) and complement fixation (CF) test results in horses from stables with silent infection (zone A)

		ELISA		
		Positive	Negative	Total
CF test	Positive	26	4	30
CF test	Negative	37	88	125
	Total	63	92	155

To investigate whether rates of seropositivity differed between premises with and without clinical cases, the proportion of horses that were CF test–positive from the stables with clinical cases was compared with the proportion obtained for horses from the stables with silent infections. Because CF test results are likely to be dependent on the time of infection, the comparison consisted of the November samples from stables with silent infections and the second set of samples taken from horses from stables with clinical cases, also obtained in November (all stables from zone A). No significant difference was observed in rates of infection for the two groups (chi-square test p>0.05).

In February–April 1999, serum samples were obtained from all the 123 horses never tested before and housed in 35 stables located in zones B and C. Seroprevalence using the IgG ELISA test was 43/120 (36%), with three inconclusive tests. CF test prevalence was 19/123 (15%), with titers ranging from 1:4 to 1:8. Fourteen stables were found positive in zone B and none in zone C (Figure 1).

Among the 123 horses tested in zones B and C, information on presence in the area during the outbreak period was available for 110, including 107 that had been present and 3 that had not. Of the 107, 40 tested positive (37%); among the 3 not present, 1 was positive. Among the 13 horses whose location during the epidemic could not be established, 2 were seropositive.

The results of serum samples collected in zones B and C at the beginning of 1999 before the growth of the vector population were compared to the results of sera from the stables without clinical cases (zone A) collected during the autumn 1998. No differences were found between the percentages of animals seropositive by ELISA (36% vs. 41%) and CF test (15% vs. 19%). For this reason, to obtain a larger sample, all data were pooled to assess the age-specific prevalence in the horse population under study.

**Figure 3:**
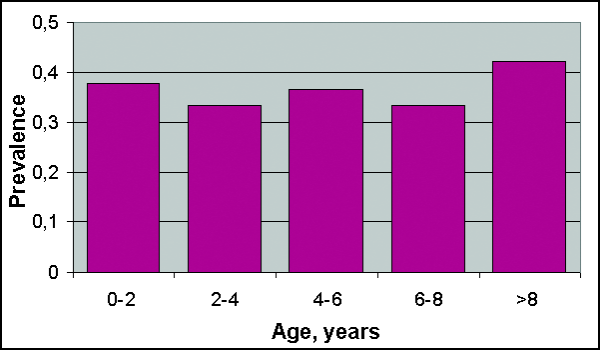
West Nile virus age-specific prevalence (n=282) in horses, Tuscany Region, 1998–1999.

A total of 282 horses were included in the analysis (Table 4) and classified according to age group and serologic status with respect to IgG ELISA (106 were positive; 9 were at the A420 cut-off value and inconclusive). No significant differences (chi-square >0.05) in the seroprevalence rate among the different age-classes were observed (Figure 3). The results of this survey confirmed a wider spread of infection, with an overall seroprevalence rate of 38% (106/282) in the affected area.

**Table 4:** Horses, classified by age class and serologic status (immunoglobulin G enzyme-linked immunosorbent assay), included in the analysis to assess age-specific prevalence^a^

Age class (yrs)	Horses tested	Inconclusive	Positive
0–2	45	3	17
2–4	63	1	21
4–6	49	1	18
6–8	30	1	10
>8	95	3	40
Total	282	9	106

^a^282 horses included because the age of 2 animals was unknown.

## Discussion

An epidemic of WNV encephalitis occurred during the late summer of 1998, among horses residing in a wetland area in the Valdinievole Valley, in the provinces of Florence and Pistoia, Tuscany. All the cases identified by the retrospective study were confirmed by serologic or virologic assays, or both.

The outbreak involved race horses and racehorse breeding stock of high economic value, and the spatial and temporal nature of this cluster aided the investigation, as did the fact that local practitioners brought the problem to the attention of veterinary public health authorities early in the the outbreak. The serologic survey demonstrated that WNV infection also occurred in premises without clinical cases and that WNV was spread over an area wider than initially detected during the epidemic.

Although the number of confirmed cases reported is small, the case-fatality rate of this 1998 Italian outbreak (6/14, 43%) is similar to the rate observed in 2000 in France (20/58, 34%), and in 1999 (23/60, 38%) and 2000 (9/25, 36%) in the United States (21,19). No differences in age were detected between fatal cases (horses that died or were euthanized) and those that recovered. Three of six fatal cases were observed in horses <6 years old, with one case 6 months old, one case 2 years old, and one case 5 years old. In the recent equine outbreak in France (21), >70% of the fatal cases occurred in horses in the 6- to 10-year (41.2%) and the 16- to 20-year (29.6%) age categories, with only one confirmed fatal case in the 1- to 5-year age category (5.9% of the fatal cases).

In our study, WNV was isolated from different parts of the central nervous system, and similar results were obtained in a horse with encephalitis euthanized during the epidemic in France in 1965 (27). In that case, WNV was isolated in cell cultures injected with material obtained from the lumbar region of the spinal cord but not with material from the cervical region of the spinal cord, the cerebrum, or the cerebellum. Although based on a very limited number of observations, we observed that the lower compartments of the brain and the spinal cord may be more infected than the cerebrum when the neurologic signs are predominant and the animal is dying. Moreover, the presence of neuronal necrosis in the brain in the absence of virus and the detection of both inflammatory lesions and virus in the lower brain stem late in the infection process suggest that the agent may have migrated from central brain areas downwards through the spinal cord (26). The low virus load in the central nervous system is in accordance with the small number of necrotic areas within the brain and the spinal cord tissues (3,26).

Two different serologic tests, which detect different classes of antibodies, can be used to distinguish between recent and past infections. CF test is used primarily to monitor IgM, although this test may not detect exclusively this class of antibodies, and the ELISA test is specific for IgG. The decline in the mean CF test antibody titer over a 1-month period (October–November 1998) in stables with clinical cases seems to be attributable to the natural decrease of CF antibody levels. The rapid decline in CF test titers may also explain the lower proportion of CF test–positive horses in the second and third groups that were tested in November and in early 1999. In the zone A stables, which had silent infections and that were investigated in November 1998, the CF test prevalence rates were similar to the rates detected in the November samples taken from the neighboring stables with clinical cases. As similar IgG ELISA prevalence rates were obtained both in these stables with silent infections located in zone A and in stables in zone B (sampled in early 1999), we assume that, apart from the observation of neurologic signs, all horses residing in the study area had the same level of exposure to the virus.

Retrospectively, serologic results suggest that the epidemic ended during the autumn of 1998, probably because further major circulation of virus was greatly reduced by the effect of the low temperature on the population density of mosquitoes. However, at present no information is available regarding the vector species involved in this outbreak.

The seasonal distribution we observed is in keeping with other studies. All previous equine and human outbreaks of WNV infection in Europe and the Mediterranean area typically have occurred between August and October (9,21), the period when the population density of culicine mosquitoes is highest. Similar observations were made in the United States in the equine outbreaks in 2000 (19), with cases identified in seven northeastern states situated at latitudes similar to that of central and northern Italy.

The differences among age-specific prevalence rates in the whole area under investigation were not significant, indicating that the horse population in the area under study had not been exposed in the previous years to WNV. The most probable hypothesis is that the virus was introduced in the Padule del Fucecchio wetlands by migratory birds during the spring 1998. Migratory birds such as storks may play an essential role in the introduction of WNV when they land in wetlands with high levels of ornithophilic mosquitoes (28). The circulation of a unique genotype in Italy in 1998, Morocco in 1999, and France in 2000 suggests that migratory birds crossing the Mediterranean Sea could be the common cause of virus emergence. The 1998 WNV outbreak in Italy was not preceded by notification of any significant deaths among wild avian species. Indeed, natural illness and death of wild birds resulting from WNV had never been reported before the 1998 episode in storks in Israel (28) and the 1999 epidemic in birds reported in the northeastern United States. Old World wild avian species may have developed co-evolutionary adaptation to various WNV genotypes; the recent North American epidemic could have occurred because the lack of avian adaptation or the greater virulence, especially for Corvidae (29), of the strain involved. The recent European WNV outbreaks were apparently not associated with bird deaths (5,21,15). The comparison of the amino acid sequence of the entire envelope protein among European WNV isolates and strains from Israel 1998 and New York 1999, known to be significantly pathogenic for some species of birds (30), showed two amino acid changes that are attenuating mutations for other flaviviruses of the Japanese encephalitis group (31). Whether viral genetic factors or ecologic factors are responsible for apparent differences in virulence for birds, horses, and humans remains unknown and merits further multidisciplinary investigation.

In 1999 and 2000, no cases of neurologic disease were recorded in horses in Tuscany, and no significant wild bird deaths or rise in human neurologic cases were detected. Unfortunately, conducting further studies to determine if any viral activity was still present in the area was not possible. The risk that WNV could remain endemic in the area because of transovarial transmission or overwinter survival of mosquitoes cannot be ruled out. However, at least in temperate regions of Eurasia, the usual pattern of WNV epidemics may be the result of virus importation by birds during their migration north to their breeding grounds (32). Still, outbreaks of WNV infection are a relatively rare event in countries where populations of wild birds regularly migrate every year from endemic areas. The reason may be that the emergence of a mosquito-borne infection always involves a series of particular ecologic conditions, including seasonal environmental factors, presence of infectious migrating hosts, ornithophilic vectors, amplifying avian hosts, and susceptible accidental hosts in the same geographic area.
